# Supplementary material for: The genome sequence of a gelechiid moth, Acompsia subpunctella Svensson, 1966 (Lepidoptera: Gelechiidae)
Source: Wellcome Open Res. 2026 Sep 4;11:581. [Version 1] doi: 10.12688/wellcomeopenres.27694.1 (PMC13662380; doi:10.12688/wellcomeopenres.27694.1)
Supplement: Peer review history

# Reviewer report: Reviewer response for version 1

**DOI:** 10.21956/wellcomeopenres.30485.r172084

**Published:** 2026-10-13

**Authors:** Bengt Hansson

**Affiliations:** 1 Lund University, Lund, Sweden

This genome note reports a high-quality genome assembly for the gelechiid moth *Acompsia subpunctella*. The authors presents a haplotype resolved assembly with haplotype 1 scaffolded into 30 chromosomal pseudomolecules, including the Z chromosome. The mitochondria was also assembled. The work contributes to Project Psyche, which aims to generate reference genomes for European butterflies and moths.

 The manuscript is clearly written and presents a valuable genomic resource. The Hi-C contact map looks good and provides convincing support for the chromosome-scale assembly. It was also excellent to include the synteny analyses identifying the Merian elements, as these results strengthen the assembly validation and place the genome in a broader comparative genomic context.

Are the datasets clearly presented in a usable and accessible format, and the assembly and annotation available in an appropriate subject-specific repository?

Yes

Are sufficient details of the sequencing and extraction, software used, and materials provided to allow replication by others?

Yes

Are the rationale for sequencing the genome and the species significance clearly described?

Yes

Are the protocols appropriate and is the work technically sound?

Yes

Reviewer Expertise:

Evolutionary biology and population genetics.

I confirm that I have read this submission and believe that I have an appropriate level of expertise to confirm that it is of an acceptable scientific standard.

# Reviewer report: Reviewer response for version 1

**Published:** 2026-09-04

**Authors:** Automated Benchmarking

**Affiliations:** 1 Wellcome Sanger Institute, Hinxton, UK

As this article is a Genome Note, Automated Benchmarking has been used to determine whether the reported assembly passes three defined checks established by the Earth BioGenome Project: the raw and assembled data are publicly available, the Earth BioGenome Project three-part metric is met, and the BUSCO score passes the stated threshold. For Acompsia subpunctella (ilAcoSubp1), all three checks passed. Data available: pass. ENA study PRJEB96678, raw-read accessions ERR15499529 and ERR15500488, and assembly accessions GCA_976986875.1 and GCA_976986855.1 are public. Earth BioGenome Project three-part metric: pass. Haplotype 1 is reported as 7.C.Q69, meeting the 6.C.Q40 reference standard. BUSCO: pass. Haplotype 1, which is proposed as the reference assembly for this species, has 99.5% complete BUSCOs (99.0% single-copy and 0.5% duplicated). It meets the target of greater than 90% complete and single-copy BUSCOs. This decision uses values reported in the Genome Note and public accession records.

Reviewer Expertise:

NA

I confirm that I have read this submission and believe that I have an appropriate level of expertise to confirm that it is of an acceptable scientific standard.
